# Vasorelaxant and Hypotensive Effects of *Galla chinensis* in Rats

**DOI:** 10.3390/ijms25147962

**Published:** 2024-07-21

**Authors:** Sujin Shin, Junkyu Park, Ho-Young Choi, Youngmin Bu, Kyungjin Lee

**Affiliations:** 1 Department of Korean Medicine, Graduate School, Kyung Hee University, Seoul 02447, Republic of Korea; sjshin04@khu.ac.kr; 2 Department of Science in Korean Medicine, Graduate School, Kyung Hee University, Seoul 02447, Republic of Korea; ojeoksan@khu.ac.kr; 3 Department of Herbal Pharmacology, College of Korean Medicine, Kyung Hee University, Seoul 02447, Republic of Korea; hychoi@khu.ac.kr (H.-Y.C.); ymbu@khu.ac.kr (Y.B.)

**Keywords:** *Galla chinensis*, Chinese gallnut, blood pressure, hypertension, hypotensive effect, vasorelaxant, endothelium, NO/cGMP pathway, cardiovascular diseases, spontaneously hypertensive rat

## Abstract

Previous studies have revealed the medicinal and therapeutic effects of *Galla chinensis*. However, no studies have focused on the antihypertensive effects of *G. chinensis*. Therefore, we aimed to determine the vasorelaxant and hypotensive effects of *G. chinensis* 50% ethanolic extract (GCE). To evaluate the vascular relaxing effect of GCE, experiments were conducted using aortic segments dissected from Sprague Dawley rats. GCE showed a vasorelaxant effect via the nitric oxide/cyclic guanosine 3′,5′-monophosphate pathway, inhibiting Ca^2+^ channels, and activating K^+^ channels. The hypotensive effects of GCE were evaluated in spontaneously hypertensive rats (SHRs). The SHRs were randomly divided into a control group and orally administered GCE group (100 or 300 mg/kg). The systolic and diastolic blood pressure decreased significantly by −19.47 ± 4.58% and −31.14 ± 7.66% in the GCE 100 mg/kg group, and −21.64 ± 2.40% and −31.91 ± 5.75% in the GCE 300 mg/kg group at 4 h after administration. Considering its vasorelaxant and hypotensive effects, our results indicate that GCE may be a valuable solution for the control of hypertension. However, further studies on the long-term administration and toxicity of GCE are required.

## 1. Introduction

Cardiovascular diseases (CVDs) affect the heart and blood circulation and impose a significant global burden of high morbidity and mortality [1]. The loss of productivity and medical expenses due to CVDs are expected to increase to $1.1 trillion by 2035 [2]. Hypertension is a critical risk factor for CVDs, and its association is stronger with a younger age of onset [3]. As the population ages, the prevalence of hypertension has increased from 594 million in 1975 to 1_·_13 billion in 2015 [4]. The World Health Organization (WHO) aims to reduce global hypertension prevalence by 25% by 2025. However, blood pressure (BP) is not adequately controlled in many patients due to poor adherence to antihypertensive treatment, the unwanted side effects of current medications, prescription errors, and unsupportive healthcare systems [5].

The interest in and demand for natural products are growing with the increasing prevalence of non-communicable diseases in aging populations [6]. Natural products offer huge potential for healthcare owing to their wide chemical and structural diversity [7]. Natural products are effective in preventing and managing CVDs because of their antioxidant, anti-hypercholesterolemic, anti-ischemic, vasodilatory, and platelet aggregation-inhibiting effects [8,9]. However, only a few natural products have been scientifically investigated for their therapeutic effects [10]. Therefore, exploring the efficacies of natural products is necessary.

*Galla chinensis,* also known as nutgall, is a gall produced on the leaves of plants of the genus *Rhus* in response to secretions by the aphid *Melaphis chinensis* (Bell Baker) [11]. Galls made from *Rhus chinensis* are of the highest quality [12]. *G. chinensis* has been used to treat diarrhea, dysentery, hemorrhage, cough, and bloody sputum [13]. Recent studies revealed a wide range of therapeutic effects for *G. chinensis*, including antibacterial, anticarcinogenic, anticancer, antithrombin, and anti-diabetic effects [12]. The main characteristic components of *G. chinensis* are tannins, phenolic acids, amino acids, and trace elements, such as Cu, Zn, Fe, and Ca [11].

Previous studies have revealed the vasorelaxant and hypotensive effects of *Rhus chinensis* [14]. However, the antihypertensive efficacy of *G. chinensis* produced from plants of the genus *Rhus* has not yet been elucidated. Therefore, we aimed to reveal the vasorelaxant effect and underlying mechanisms of *G. chinensis* in Sprague Dawley (SD) rats, and investigate its antihypertensive effects in spontaneously hypertensive rats (SHRs).

## 2. Results

### 2.1. High-Performance Liquid Chromatography (HPLC) Analysis of G. chinensis

**Figure 1:**
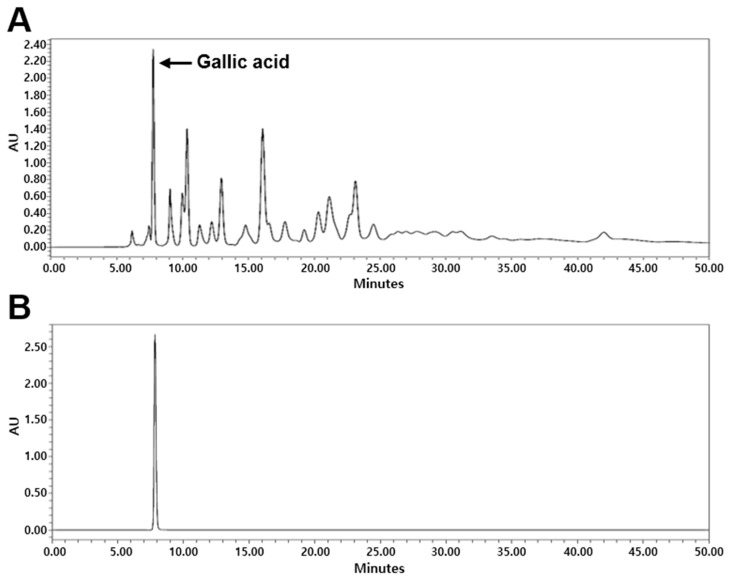
High-performance liquid chromatography (HPLC) analysis of (**A**) *Galla chinensis* 50% ethanol extract (GCE) and (**B**) gallic acid. AU, Absorbance units.

The content of gallic acid, which is the main component of the *G. chinensis* 50% ethanol extract (GCE), was determined using HPLC analysis (Figure 1). The content of gallic acid in GCE was 5.56%.

### 2.2. Vascular Relaxation Induced by GCE on the Endothelium-Intact or -Removed Aorta

**Figure 2:**
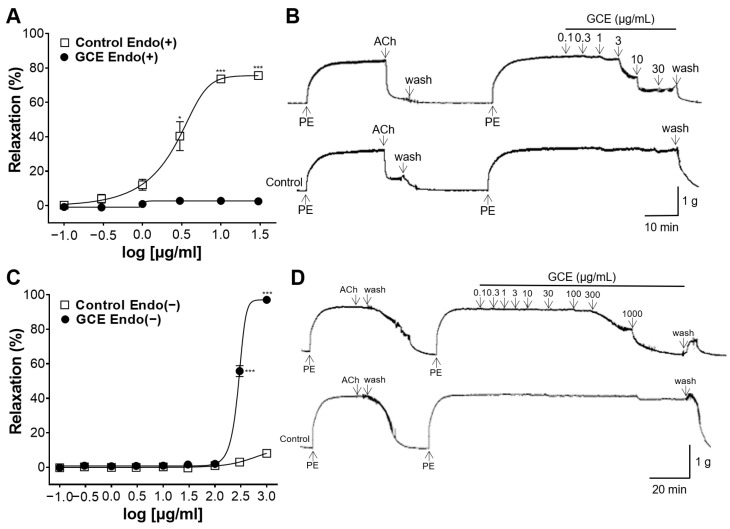
*Galla chinensis* 50% ethanol extract (GCE)-induced concentration-dependent relaxation of the endothelium-intact [Endo(+)] or -removed [Endo(−)] aorta. To confirm the integrity of endothelium, aortas pre-constricted by phenylephrine (PE) were administered with acetylcholine (ACh). (**A**) Relaxation response to cumulative doses of GCE and (**B**) representative tracing. Data are shown as the means ± standard error of the mean (SEM) (*n* = 4–5). * *p* < 0.05, *** *p* < 0.001 vs. control.

To elucidate the vasorelaxant effect of GCE, the concentration responses to GCE were evaluated in the endothelium-intact or -removed aorta. GCE exerted a concentration-dependent vascular relaxing effect on both endothelium-intact or -removed rings; however, their EC_50_ values were different (Figure 2). In the endothelium-intact rings, the EC_50_ value was 2.80 ± 1.13 μg/mL, and the E_max_ value was 75.59 ± 0.58% at 30 μg/mL (Figure 2A,B). In the endothelium-removed rings, the EC_50_ value was 285.70 ± 32.43 μg/mL, and the E_max_ value was 97.09 ± 1.20% at 1000 μg/mL (Figure 2C,D). These results indicate that the vasodilatory action of GCE occurred in both an endothelium-dependent and -independent manner.

### 2.3. Vascular Relaxation Induced by GCE on Aortas Incubated with Nitric Oxide (NO) Synthase Inhibitor, Cyclooxygenase (COX) Inhibitor, Soluble Guanylate Cyclase (sGC) Inhibitor or cGMP Inhibitor

**Figure 3:**
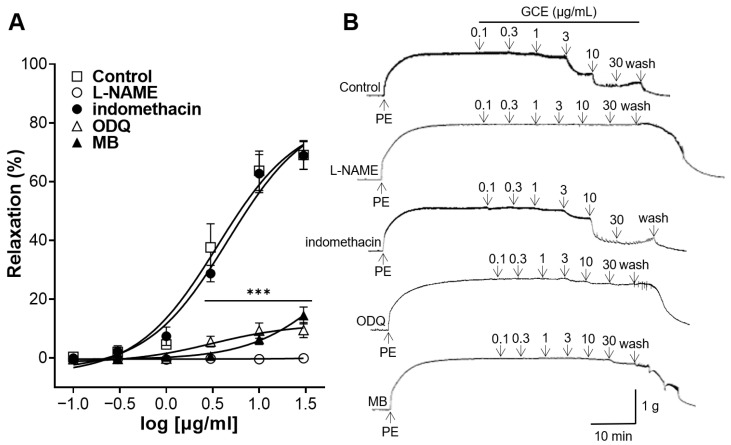
*Galla chinensis* 50% ethanol extract (GCE)-induced concentration-dependent relaxation of aortic rings pre-incubated with N^G^-nitro-L-arginine methyl ester (L-NAME), indomethacin, 1H-[1,2,4]Oxadiazolo[4,3-a]quinoxalin-1-one (ODQ) or methylene blue (MB). Rings were constricted by phenylephrine (PE) before the GCE-induced relaxation. (**A**) Relaxation response to cumulative doses of GCE and (**B**) representative tracing. Data are shown as the means ± SEM (*n* = 5). *** *p* < 0.001 vs. control.

To confirm whether GCE-induced relaxation is associated with the activation of NO synthase or COX, the aortic rings were pre-treated with N^G^-nitro-L-arginine methyl ester (L-NAME), an inhibitor of NO synthase, or indomethacin, a COX inhibitor. L-NAME significantly reduced the GCE-induced relaxation; however, indomethacin showed no significant difference from the control group without the pre-treatment (Figure 3). These results suggest that the mechanism of the vasorelaxation of GCE was related to NO synthase.

To confirm whether GCE-induced relaxation is associated with sGC or cGMP, thoracic aortas were pre-incubated with 1H-[1,2,4]Oxadiazolo[4,3-a]quinoxalin-1-one (ODQ), an inhibitor of sGC, or methylene blue (MB), a cGMP inhibitor. ODQ and MB significantly inhibited the GCE-induced relaxation (Figure 3). These results suggest that the mechanism of the vasorelaxation of GCE was mediated by sGC and cGMP.

### 2.4. Inhibitory Effect of GCE on Vasoconstriction Induced by Ca^2+^

**Figure 4:**
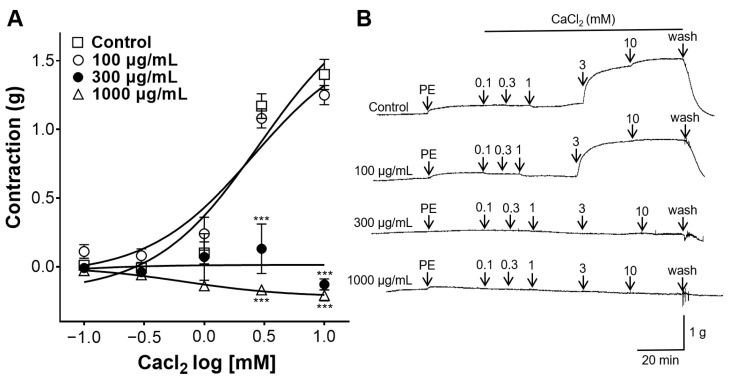
Inhibitory effect of *Galla chinensis* 50% ethanol extract (GCE) on vasoconstriction of endothelium-intact aortic rings induced by calcium chloride (CaCl_2_). Rings were pre-treated with phenylephrine (PE) before the vasoconstriction by CaCl_2_. (**A**) Contraction response to cumulative doses of GCE and (**B**) representative traces. Data are shown as the means ± SEM (*n* = 4–5). *** *p* < 0.001 vs. control.

To confirm whether the GCE-induced relaxation was associated with receptor-operated Ca^2+^ channels, the contraction of the aortic rings was induced by phenylephrine (PE), and calcium chloride (CaCl_2_) was cumulatively added. GCE (300 and 1000 μg/mL) significantly attenuated the Ca^2+^-induced contraction in the endothelium-intact rings. The maximal constriction induced by CaCl_2_ (10 mM) was 1.40 ± 0.11 g, 1.25 ± 0.07 g, −0.13 ± 0.04 g, and −0.21 ± 0.04 g in the absence and presence of GCE (100, 300, and 1000 μg/mL), respectively (Figure 4).

### 2.5. Vascular Relaxation Induced by GCE on Aortas Pre-Treated with K^+^ Channel Blockers

**Figure 5:**
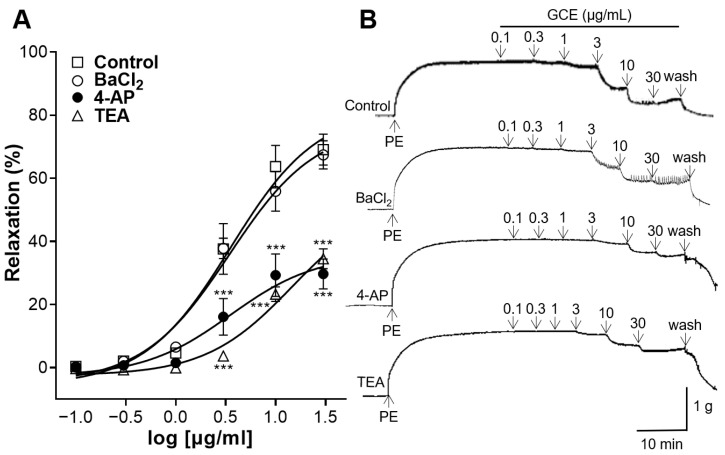
*Galla chinensis* 50% ethanol extract (GCE)-induced concentration-dependent relaxation of the aortic rings pre-treated with barium chloride (BaCl_2_), 4-aminopyridine (4-AP), or tetraethylammonium (TEA). Rings were constricted with phenylephrine (PE) before the GCE-induced relaxation. (**A**) Relaxation response to cumulative doses of GCE and (**B**) representative tracing. Data are shown as the means ± SEM (*n* = 4–6). *** *p* < 0.001 vs. control.

To confirm whether GCE-induced relaxation is associated with K^+^ channels, the aortic rings were incubated with the inward rectifier K^+^ channel blocker barium chloride (BaCl_2_), the voltage-dependent K^+^ channel blocker 4-aminopyridine (4-AP), or the large-conductance Ca^2+^-activated K^+^ channel blocker tetraethylammonium (TEA). 4-AP and TEA significantly reduced GCE-induced relaxation; however, BaCl_2_ showed no significant difference compared with the control group without the pre-treatment (Figure 5).

### 2.6. Inhibitory Effect of GCE on Vasoconstriction Induced by Angiotensin II

**Figure 6:**
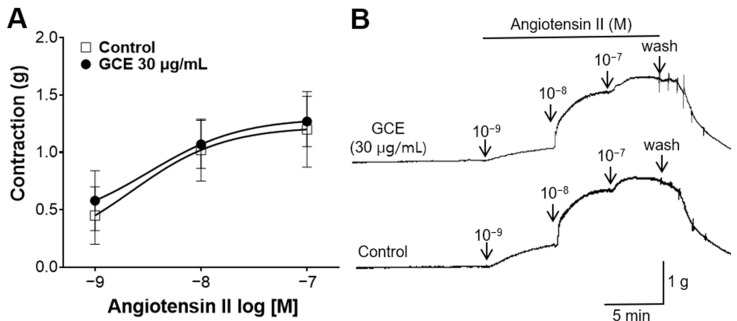
Effects of *Galla chinensis* 50% ethanol extract (GCE) on aortas constricted by angiotensin II. (**A**) Contraction response to cumulative doses of GCE and (**B**) representative traces. Data are shown as the means ± SEM (*n* = 5).

To confirm whether the GCE-induced relaxation is associated with angiotensin II, the contraction of the aortic rings was induced by angiotensin II. GCE (30 μg/mL) showed no significant difference compared with the control group without the pre-treatment (Figure 6).

### 2.7. Hypotensive Effect of GCE

**Figure 7:**
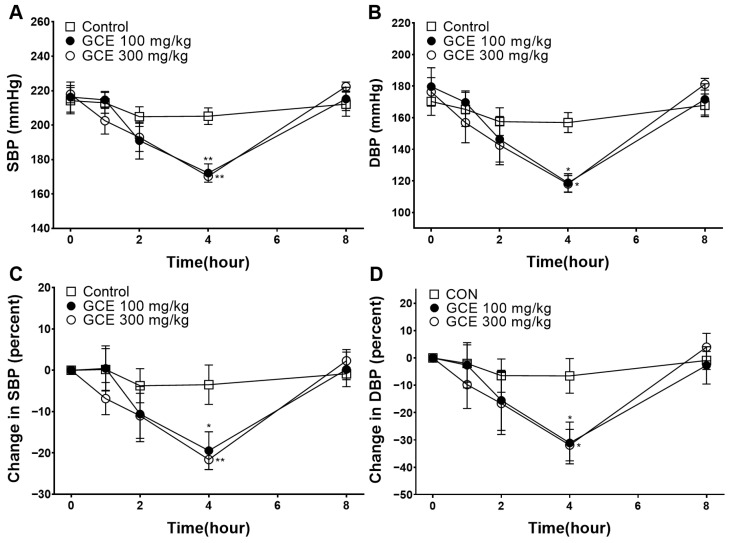
Hypotensive effect of *Galla chinensis* 50% ethanol extract (GCE). Blood pressure of spontaneously hypertensive rats was measured after GCE (100 and 300 mg/kg) administration. (**A**) Systolic blood pressure (SBP), (**B**) diastolic blood pressure (DBP), (**C**) percent changes in SBP, (**D**) percent changes in DBP. Data are shown as the means ± SEM (*n* = 6–7). * *p* < 0.05, ** *p* < 0.01 vs. control.

To determine the effect of lowering the blood pressure in the SHRs, GCE (100 and 300 mg/kg) was administered. The lowest blood pressure was measured 4 h after administration in the GCE 100 and 300 mg/kg group, respectively (Figure 7). The percentage reductions in the systolic blood pressure (SBP) and diastolic blood pressure (DBP) were −19.47 ± 4.58% and −31.14 ± 7.66% in the GCE 100 mg/kg group at 4 h. The percentage reductions in the SBP and DBP were −21.64 ± 2.40% and −31.91 ± 5.75% in the GCE 300 mg/kg group at 4 h.

## 3. Discussion

Blood pressure control is essential for preventing CVDs, and one of the key contributors to increased blood pressure is endothelial dysfunction [15,16]. The endothelium senses the shear stress acting on the vessel wall and regulates the vessel tone by releasing a variety of vasoactive factors [17]. Endothelial cells synthesize a variety of substances for vascular homeostasis, including NO, the main vascular relaxing factor, and are the primary targets for vasorelaxant action [18,19]. Therefore, to investigate the applicability of GCE in managing hypertension, its vasorelaxant effect on the contractility of isolated rat thoracic aortas was investigated. Since the function of aortic endothelial cells is relatively impaired in SHRs, this results in the decreased production of vasoactive factors [20]. We used thoracic aortas from healthy SD rats rather than SHRs to clearly understand the mechanisms involved in this study.

To reveal the vasorelaxant effects and mechanisms related to endothelial cells, GCE was cumulatively added to endothelium-intact or endothelium-removed aortas. Our experiment showed that GCE resulted in a concentration-dependent vasorelaxant response, and the concentrations at which significant vasorelaxant effects occurred differed depending on the presence of endothelial cells. GCE induced a significant relaxation response with EC_50_ values of 2.80 ± 1.13 μg/mL in the endothelium-intact aortas and 285.70 ± 32.43 μg/mL in the endothelium-absent aortas. Our experimental results showed that the vasodilatory mechanism of GCE primarily relies on the endothelial cells at relatively low concentrations, and the mechanisms unrelated to endothelial cells also contribute at high concentrations.

To further elucidate the endothelial cell-related mechanisms involved in the vasorelaxant effects of GCE, additional experiments were conducted. NO is synthesized in the endothelium by NO synthase and signals vascular smooth muscle cells (VSMCs) to induce vasorelaxation through the NO-sGC-cGMP pathway [21]. In addition, prostacyclin (PGI_2_) is synthesized in the endothelium and induces vasodilation via the cyclic adenosine monophosphate-dependent pathway [18]. In our study, L-NAME (an NO synthase inhibitor) and indomethacin (a COX inhibitor) were used to investigate whether the vasorelaxant mechanism of GCE is mediated by NO or COX. The L-NAME pre-treatment prevented GCE-induced relaxation; however, there was no significant difference in the vascular relaxation effect of the indomethacin pre-treatment. The next group of experiments was performed by pre-treating the aorta with ODQ (sGC inhibitor) or MB (cGMP inhibitor). The ODQ or MB pre-treatment also prevented GCE-induced relaxation. Thus, our study indicates that the vasodilatory effect of GCE is a consequence of the activation of the NO-sGC-cGMP pathway, but is independent of PGI_2_.

Vascular tension in the small arteries and arterioles is mainly regulated by Ca^2+^ and K^+^ channels in the plasma membrane of VSMCs [22]. The contraction of blood vessels is regulated by extracellular Ca^2+^ influx via Ca^2+^ channels because of the underdevelopment of the sarcoplasmic reticulum in VSMCs [22]. Increased intracellular Ca^2+^ concentrations induce actin–myosin cross-bridge formation and initiate VSMC contraction [23]. In this study, the involvement of receptor-operated Ca^2+^ channels in the vasodilatory effect of GCE was investigated. GCE (300, and 1,000 μg/mL) significantly attenuated Ca^2+^-induced contraction compared with the control group. High concentrations of GCE exhibit vasorelaxant effects via endothelium-independent mechanisms. Therefore, our study indicates that the receptor-operated Ca^2+^ channel-blocking effect of GCE works at high concentrations, and is a mechanism independent of endothelial cells.

Additionally, the involvement of K^+^ channels in the vasodilatory action of GCE on the aortic rings was studied. The relaxation of VSMCs is regulated by changes in ion flux and membrane potential [23]. Various subtypes of K^+^ channels determine the membrane potential, including inward rectifier K^+^ channels, voltage-dependent K^+^ channels, and large-conductance Ca^2+^-activated K^+^ channels [24]. The opening of these K^+^ channels results in the hyperpolarization of the cell membrane and induces vasorelaxation [25]. We used selective blockers of these channels to study their involvement in GCE-induced vasorelaxation. 4-AP (a voltage-dependent K^+^ channel blocker) and TEA (a large-conductance Ca^2+^-activated K^+^ channel blocker) significantly reduced GCE-induced relaxation; however, the pre-treatment with BaCl_2_ (an inward rectifier K^+^ channel blocker) showed no significant difference. Therefore, K^+^ channels, especially voltage-dependent and large-conductance Ca^2+^-activated K^+^ channels, mediated the vasorelaxant effects induced by GCE.

The renin–angiotensin system (RAS) is a hormonal pathway that controls blood volume and systemic vascular resistance; it commences with angiotensinogen, which is converted into angiotensin I by renin [26]. Subsequently, angiotensin I is cleaved into angiotensin II, and the activation of the angiotensin II type 1 receptor induces vasoconstriction and increases sympathetic nervous system activity, resulting in elevated blood pressure [27]. Some natural products have been shown to inhibit the vasoconstrictive action of angiotensin II [28,29]. We investigated the effect of GCE on the action of angiotensin II. Administering GCE (30 μg/mL) resulted in no significant difference in the degree of contraction of the aorta constricted with angiotensin II compared with the control group. Our study suggests that GCE does not affect the vasoconstrictive action of angiotensin II. However, further research is needed for the effects of GCE on RAS, including the inhibitory effects of angiotensin converting enzyme, synthesis of angiotensin II, and inhibition of renin.

Comprehensively, GCE exerted a vascular relaxing effect via the NO/cGMP pathway by blocking Ca^2+^ channels and activating voltage-dependent and large-conductance Ca^2+^-activated K^+^ channels in the thoracic aorta of SD rats. However, additional experiments on the vasodilation effects of GCE in the smaller arterioles are needed to further clarify that the vasorelaxant effect of GCE helps to control blood pressure.

Finally, the blood pressure-lowering effect of orally administered GCE in SHRs was investigated. SHRs are commonly used as experimental models of hypertension due to the similarity of hypertension pathology between SHRs and humans, and the comparable hypotensive effects observed with antihypertensive agents in both species [30]. In general, SHRs over 16 weeks of age are hypertensive, with an SBP of 180–200 mmHg or higher [30,31]. In our study, GCE was orally administered to 40-week-old SHRs with an average SBP of 200–210 mmHg and DBP of 170–180 mmHg. The greatest reduction rates in the SBP and DBP (−21.64 ± 2.40% and −31.91 ± 5.75%, respectively) were measured at 4 h after administration in the GCE 300 mg/kg group. In the GCE 100 mg/kg group, the reduction rates in the SBP and DBP (−19.47 ± 4.58% and −31.14 ± 7.66%, respectively) were measured 4 h after administration. Male SHRs are commonly used as an established model of hypertension; however, there are sex differences, as there are in humans, such as hypertension developing more rapidly and becoming more severe in males than in females [32]. Previous studies have shown sex differences in the activation of pathways that contribute to hypertension [33]. Therefore, further studies are required to investigate the effects of GCE on female SHRs.

In our previous study, we discussed the vascular relaxing and blood pressure-lowering effects of *Rhus chinensis* leaf ethanol extract (NTE). *G. chinensis* is a gall produced on the plants of the genus *Rhus* in response to secretions by the aphid *M. chinensis*, and because its medicinal properties differ from those of the *Rhus chinensis*, *G. chinensis* has been used as a separate traditional remedy. Our experimental results showed that NTE and GCE showed vasorelaxant effects, but they differed in their mechanisms. Compared to NTE, GCE exerts a Ca^2+^ channel blocking effect, but did not inhibit the contractile action of angiotensin II. In addition, when comparing the hypotensive effect in SHRs, NTE showed no significant difference from the control group at a dose of 300 mg/kg; however, GCE showed a significant blood pressure-lowering effect at 100 and 300 mg/kg.

Considering its vasorelaxant and hypotensive effects, GCE may be a valuable source for the control of hypertension. Previous studies have reported that the LD_50_ of *G. chinensis* was higher than 5000 mg/kg body weight (bw) per day in an acute toxicity test, and the no-observed-effect level was lower than 1500 mg/kg bw per day [11,34]. However, further studies are needed to examine the effects and toxicities of long-term drug administration.

## 4. Materials and Methods

### 4.1. Preparation of the Extract

*G. chinensis* was purchased from CK Pharm Co., Ltd (Seoul, Republic of Korea). *G. chinensis* was extracted by boiling with 10-fold amount of 50% ethanol for 2 h at a temperature of 70 ± 2 °C. The GCE was then filtered twice with filter paper No. 2 (Hyundai Micro, Seoul, Republic of Korea) and freeze-dried. The yield of the GCE was 60.27%.

### 4.2. HPLC Analysis of GCE

Experiments were performed using Waters e2695 Alliance HPLC system (Waters Corp., Milford, MA, USA) equipped with a 2998 photodiode array detector (PDA). Chromatographic separation was carried out on a C18 column (250 × 4.6 mm, 5 µm) (Fortis Technologies Ltd., Cheshire, UK). Two solvents were used for the isocratic mobile phase: 1% acetic acid and acetonitrile (80:20, *v*/*v*). The flow rate was set at 0.4 mL/min, with column temperature maintained at 25 °C, and detection was performed at UV 290 nm. GCE (10 mg/mL) and gallic acid standard (1 mg/mL) were filtered twice through a 0.45 µm PVDF syringe filter (Korea Ace Science, Seoul, Republic of Korea). The standard was serially diluted, and HPLC chromatograms were obtained. Empower 2 software (Waters Corp., Milford, MA, USA) was used for data acquisition.

### 4.3. Animals

Male 6–7-week-old SD rats (Daehan Biolink, Eumseong-gun, Republic of Korea), and male 40-week-old SHRs (SLC, Inc., Shizuoka, Japan) were used in this study. Animals were housed in a controlled environment maintained at a temperature of 22 ± 2 °C, humidity 45–65%, and 12/12 h light/dark cycle. The animals were given free access to both feed and tap water. All experiments complied with the Animal Welfare Guidelines and were approved by the Animal Experiment Ethics Committee of Kyung Hee University (KHSASP-23-506).

### 4.4. Chemicals and Buffer Preparation

Acetic acid, BaCl_2_, glucose, magnesium sulfate (MgSO_4_), monobasic potassium phosphate (KH_2_PO_4_), potassium chloride (KCl), sodium chloride (NaCl), sodium hydrogen carbonate (NaHCO_3_), CaCl_2_, and urethane were purchased from Daejeong Chemical and Gold (Siheung-si, Republic of Korea). Acetonitrile was purchased from JT Baker (Phillipsburg, NJ, USA). Dimethyl sulfoxide (DMSO) was purchased from Junsei (Tokyo, Japan). PE, acetylcholine (ACh), indomethacin, MB, ethyleneglycol-bis(2-aminoethylether)-N,N‚N′,N′-tetraacetic acid (EGTA), and angiotensin II were purchased from Sigma Aldrich (St. Louis, MO, USA). L-NAME, 4-AP, and TEA were purchased from Wako Pure Chemical Industries (Osaka, Japan). ODQ was purchased from the Tokyo Chemical Industry (Tokyo, Japan).

The composition of Krebs–Henseleit (KH) buffer used in this study was as follows: 118.0 mM NaCl, 4.7 mM KCl, 2.5 mM CaCl_2_, 1.2 mM MgSO_4_, 1.2 mM KH_2_PO_4_, 25.0 mM NaHCO_3_, and 11.1 mM of glucose.

### 4.5. Preparation of Aortic Rings and Measurement of the Tension

#### 4.5.1. General Experimental Procedures 

The vasorelaxant effect was measured as described previously [35]. SD rats were subjected to anesthesia (urethane, intraperitoneal injection of 1.2 g/kg bw). The thoracic aorta was extracted, was cut into 2–3 mm long segments, and were hung between hooks in glass chambers filled with KH buffer, which was continuously supplied with a mixed gas (95% O_2_ and 5% CO_2_), and maintained at 37 °C. Multiple aortic rings were obtained from one animal and used in subsequent experiments. The number *n* of data points in the figure indicates the number of vessel rings. Aortic rings were never used in more than one experiment. The aortic rings were stabilized with a passive tension of 1.0 g in the chamber for 40–50 min, and washed with fresh KH buffer every 10 min. GCE was dissolved in DMSO, and was added to the KH buffer in the chamber according to each experimental protocol. The DMSO concentration used was less than 1%. The control group was treated with the same dose of DMSO as GCE.

#### 4.5.2. Vascular Relaxation Induced by GCE on the Endothelium-Intact or -Removed Aortic Rings

To confirm the integrity of endothelium, the aortic rings pre-constricted with PE (1 μM) were subjected to ACh (10 μM) administration. Endothelium-intact rings were identified by a relaxant response to ACh of more than 85%. To process the endothelium-removed rings, the endothelium was manually rubbed by cotton swabs. Endothelium-removed rings were identified by a relaxant response to ACh of less than 10%. After confirming endothelial integrity, rings were washed with KH buffer and were constricted again with PE (1 μM). After blood vessels reached the plateau, cumulative concentrations of GCE were each applied to the endothelium-intact or -removed aortic rings.

#### 4.5.3. Vascular Relaxation Induced by GCE on Aortic Rings Pre-Treated with L-NAME, Indomethacin, ODQ, or MB

Thoracic aortic rings were pre-incubated with L-NAME (100 μM), indomethacin (10 μM), ODQ (10 μM) or MB (10 μM) for 20 min and were constricted with PE (1 μM). Cumulative concentrations of GCE were added to each chamber after blood vessels reached the plateau.

#### 4.5.4. Inhibitory Effect of GCE on Vasoconstriction Induced by Ca^2+^

Thoracic aortic rings were stabilized in Ca^2+^-free KH buffer containing EGTA (1 mM), and were pre-incubated with GCE (100, 300, and 1000 μg/mL) for 20 min, and PE (1 μM) for another 20 min. PE was administered to activate the receptor-operated Ca^2+^ channel. Then, cumulative concentrations of CaCl_2_ were each applied to the endothelium-intact aortic rings.

#### 4.5.5. Vascular Relaxation Induced by GCE on Aortic Rings Pre-Treated with K^+^ Channel Blockers

Thoracic aortic rings were pre-incubated with BaCl_2_ (10 μM), 4-AP (1 mM), or TEA (1 mM) for 20 min and were constricted with PE (1 μM). Cumulative concentrations of GCE were added to each chamber after blood vessels reached the plateau.

#### 4.5.6. Inhibitory Effect of GCE on Vasoconstriction Induced by Angiotensin II

Thoracic aortic rings were pre-incubated with GCE (30 μg/mL) for 20 min. Then, cumulative concentrations of angiotensin II were added to induce contraction.

### 4.6. Measurement of Blood Pressure in SHR

The SHRs were randomly allocated to either the control group (orally administered distilled water) or the GCE administration group (orally administered 100 mg/kg bw and 300 mg/kg bw dissolved in distilled water). SBP and DBP were measured before administration and at 1, 2, 4, and 8 h after administration by the tail-cuff method as described previously [35].

### 4.7. Statistical Analysis

Non-linear regression, specifically a sigmoidal dose–response model, was used for curve fitting to determine the relationship between the cumulative dose and response. Experimental data are presented as the mean ± SEM. Statistical analysis was performed using multiple unpaired *t*-tests and two-way analysis of variance (ANOVA), followed by Dunnett’s post hoc test. Statistical significance was defined as *p* < 0.05.

## 5. Conclusions

GCE exerts a vascular relaxant effect through the NO/cGMP pathway, blocking receptor-operated Ca^2+^ channels, and activating voltage-dependent and large-conductance Ca^2+^-activated K^+^ channels. In addition, the oral administration of GCE significantly reduced the blood pressure in SHRs. Considering its vasorelaxant and hypotensive effects, GCE may be a valuable source for the control of hypertension. However, further research is required to explore the effects of the long-term administration and toxicity rates of GCE.

## Data Availability

The data presented in this study are available from the corresponding author upon request.
